# Identification of Achaete-scute complex-like 1 (ASCL1) target genes and evaluation of DKK1 and TPH1 expression in pancreatic endocrine tumours

**DOI:** 10.1186/1471-2407-9-321

**Published:** 2009-09-10

**Authors:** Térèse A Johansson, Gunnar Westin, Britt Skogseid

**Affiliations:** 1 Department of Medical Sciences, Uppsala University, Uppsala University Hospital, SE-751 85 Uppsala, Sweden; 2 Department of Surgical Sciences, Uppsala University, Uppsala University Hospital, SE-751 85 Uppsala, Sweden

## Abstract

**Background:**

*ASCL1 *role in pancreatic endocrine tumourigenesis has not been established. Recently it was suggested that ASCL1 negatively controls expression of the Wnt signalling antagonist *DKK1*. Notch signalling regulates expression of TPH1, the rate limiting enzyme in the biosyntesis of serotonin. Understanding the development and proliferation of pancreatic endocrine tumours (PETs) is essential for the development of new therapies.

**Methods:**

*ASCL1 *target genes in the pancreatic endocrine tumour cell line BON1 were identified by RNA interference and microarray expression analysis. Protein expressions of selected target genes in PETs were evaluated by immunohistochemistry.

**Results:**

158 annotated *ASCL1 *target genes were identified in BON1 cells, among them DKK1 and TPH1 that were negatively regulated by ASCL1. An inverse relation of ASCL1 to DKK1 protein expression was observed for 15 out of 22 tumours (68%). Nine tumours displayed low ASCL1/high DKK1 and six tumours high ASCL1/low DKK1 expression. Remaining PETs showed high ASCL1/high DKK1 (n = 4) or low ASCL1/low DKK1 (n = 3) expression. Nine of twelve analysed PETs (75%) showed TPH1 expression with no relation to ASCL1.

**Conclusion:**

A number of genes with potential importance for PET tumourigenesis have been identified. *ASCL1 *negatively regulated the Wnt signalling antagonist *DKK1*, and *TPH1 *expression in BON1 cells. In concordance with these findings DKK1 showed an inverse relation to ASCL1 expression in a subset of PETs, which may affect growth control by the Wnt signalling pathway.

## Background

Pancreatic endocrine tumours (PETs) are derived from the embryologic endoderm and accounts for 1-2% of pancreatic cancer. The only currently curative therapy for patients with PETs is surgical resection. PETs occur sporadically or are familial in nature, caused by germ line mutations in the Multiple endocrine neoplasia 1 (*MEN1*) or von Hippel-Lindau (*VHL*) tumour suppressor genes. Understanding the molecular pathways that control PET development and proliferation are essential for possible development of novel therapies.

The basic helix loop helix (bHLH) transcription factor Achaete-scute complex homolog 1 (*Ascl1*) has been shown to play important regulatory roles in adrenal medullary chromaffin cells [1], thyroid parafollicular C-cells [2] and pulmonary endocrine cells [3]. *Ascl1 *is tightly controlled by the Notch signalling pathway in the developing pancreas and governs the exocrine versus endocrine cell fate decision [4]. Forced Notch activation expands the pool of undifferentiated precursor cells and inhibits the initial emergence of endocrine cells and the following exocrine differentiation [5,6], whereas disruption of Notch signalling results in precocious endocrine differentiation [4]. The active form of Notch, NICD, induces the expression of Hairy and enhancer of split 1 (*HES1*) which in turn antagonises the expression of bHLH genes such as *ASCL1*, with subsequent inhibition of progenitor cell differentiation [7].

We have recently reported that ASCL1 is invariably expressed in PETs, and suggested that the observed lack of nuclear HES1 might contribute to the expression of ASCL1 in these tumours [8]. In lung cancer cells ASCL1 negatively regulates the expression of Dickkopf homologue 1 (*DKK1*) [9], an antagonist of the Wnt/β-catenin signalling pathway which is involved in the development of the exocrine pancreas [10] and in pancreatic beta cell proliferation [11]. Furthermore, overexpression of NOTCH1 in the human pancreatic endocrine tumour cell line BON1 leads to inhibition of ASCL1 expression, induction of HES1, reduced levels of endocrine markers such as synaptophysin, and also to major repression of TPH1 [12], the rate limiting enzyme in serotonin biosynthesis. Serotonin is together with other hormones implicated to cause the carcinoid syndrome.

Here we report on *ASCL1 *target genes in BON1 cells transfected with *ASCL1 *siRNA. In addition, the relation of DKK1 and TPH1 protein expression to ASCL1 expression is studied in sporadic and familial (MEN 1) PETs.

## Methods

### Cell culture

The polyclonal BON1 cell line (a kind gift from Dr. J. C. Thompson, Department of Surgery, University of Texas Medical Branch, USA) was grown in 1:1 mixture of F12K (Invitrogen, Life Technologies, Carlsbad, USA) and DMEM (SVA, Uppsala, Sweden) medium supplemented with 5% foetal bovine serum. The cells were grown at 37°C in a humidified 5.0% CO_2_/air atmosphere. siRNA transfections were performed at 80% confluence. The BON1 cell line is one of few human pancreatic endocrine tumour cell lines available [13].

### Immunofluorescent microscopy

BON1 cells were seeded on glass cover slips and fixed in 3.7% formaldehyde in phosphate-buffered saline (PBS) (Sigma Aldrich, St Louis, USA) for 30 min, and washed with PBS. The cells were permeabilised in 0.2% Triton X-100 (Sigma) in PBS for 5 min, washed again in PBS, and incubated in 5% foetal bovine serum in PBS for 60 min at room temperature. Primary as well as secondary antibodies were diluted in PBS containing 5% FBS. Cells were incubated with anti-ASCL1 mouse antibody (BD Biosciences, San Jose, USA) or anti-HES1 goat antibody (Santa Cruz Biotechnology, Santa Cruz, USA) followed by FITC-labelled secondary antibodies and TRITC-labelled phalloidin with a washing step in between. The cover slips were mounted on object slides by the use of Vectashield with DAPI (Vector laboratories, Burlingame, USA). Cells were photographed by an Axiocam HRm camera employing the Axiovision imaging software using a 63× plan-apochromat objective and a Zeiss Axioplan2 microscope (Carl Zeiss Inc., Oberkochen, Germany).

### siRNA transfection

The two siRNAs were pre-designed (Ambion, USA, ID 114405 and AM4635). 5'-CGCGUUAUAGUAACUCCCATT and 5'-UGGGAGUUACUAUAACGCGTG (siRNA/A) and 5'-AGUACUGCUUACGAUACGGTT and 5'-TTUCAUGACGAAUGCUAUGCC (Control siRNA). Transfections were performed with 10-30 nmol siRNA in 12 well plates (80 0000 cells/well) using the jetSI-ENDO transfection reagent (Poly-Plus-Transfection SAS, Illkirch, France) according to the manufacturer's protocol. Samples were not pooled for downstream applications.

### RNA isolation and cDNA synthesis

Cells were harvested 72 hours after transfection and total RNA was extracted using TriZol Reagent (Invitrogen) according to manufacturer's instructions. The RNA concentration and quality were assessed using the Agilent Bioanalyser (Agilent Technologies, Palo Alto, USA). The RNeasy Mini Kit (Qiagen, Holden, Germany) was used to further purify the RNA samples. cDNA was synthesised from 1 μg of total RNA using the High Capacity cDNA Archive Kit (Applied Biosystems, Foster City, USA) according to the manufacturer's instructions.

### Quantitative real-time PCR (qPCR)

Relative mRNA expression was determined by qPCR, and compared to positive controls comprising lung carcinoid cell lines H727 and H720 (CRL-5815 and CRL-5838, LGC Promochem, Middlesex, UK. Data not shown). Commercially available primer and probe sets were used and measured against standard curves generated from dilution series of cDNA from cell lines H727, H720 and BON1. The following primers/probe mixes were used: *ASCL1*; Hs00269932_m1, *TCF3*; Hs01016249_m1, *DLL1*; Hs 00194509_m1, *SYP*; Hs00300531_m1, *TPH1*; Hs00188220_m1, and *DKK1*; Hs00183740_m1 (Applied Biosystems). Reactions were performed and analysed using an Applied Biosystems PRISM 7700 Sequence Detector. Standard cycling conditions were used. Triplicate of each cDNA was used and each assay was performed twice. The gene-specific signals were normalised to expression of *ACTB *and *PPIA *endogenous control genes (primer/probe mix 4333762F and 4333763T).

### Western blotting

Protein extracts for Western blotting were prepared by lysing the cells in RIPA buffer (Sigma-Aldrich) supplemented with complete protease inhibitor cocktail (Roche Diagnostics, Basel, Switzerland). Protein sample from each transfected well was separated in 12% SDS-polyacrylamide gradient gels (BioRad, Hercules, USA), transferred to PVDF membranes (GE Healthcare Europe GmbH, Uppsala, Sweden) and blocked with SuperBlock Blocking Buffer (Pierce Biotechnology, Rockford, USA) overnight at 4°C. The membranes were incubated with anti-ASCL1 monoclonal antibody (BD Biosciences) or anti-α- Tubulin monoclonal antibody (Santa Cruz Biotechnology) for 2 h. After briefly washing with PBS containing 0.1% Tween 20, the filters were incubated for 1 h with a secondary goat anti mouse antibody conjugated to horseradish peroxidase (1:5000 dilution). The filters were washed and developed using the Super Signal West femto kit (Pierce Biotechnology).

### Microarray analysis

RNAs from successful siRNA transfection experiments where used for microarray expression analysis. The GeneChips, Human Genome U133 Plus 2.0 (Affymetrix, Santa Clara, USA) was used for the analysis. 100 nanograms of total RNA from each sample were used to prepare biotinylated fragmented cRNA using the two-cycle cDNA synthesis part. GeneChip were hybridised for 16 hours in a 45°C incubator, rotated at 60 rpm according to the GeneChip Expression Analysis Technical Manual (Rev. 5, Affymetrix). The arrays were washed and stained using the Fluidics Station 450 and finally scanned using the GeneChip Scanner 3000 7 G.

### Bioinformatics

Differentially regulated genes were determined by calculating the fold change between the nonspecific siRNA transfected cell samples and the siRNA-ASCL1 transfected samples. Subsequent analysis of the gene expression data was carried out in the freely available statistical computing language R http://www.r-project.org using packages available from the Bioconductor project http://www.bioconductor.org. The raw data was normalised using the robust multi-array average (RMA) [14] background-adjusted, normalized and log-transformed summarised values as first suggested by Li and Wong in 2001 [15]. In order to search for the differentially expressed genes between the samples from the different groups an empirical Bayes moderated *t *test was then applied [16], using the 'limma' package [17]. To address the problem with multiple testing, the *p*-values were adjusted according to Benjamini and Hochberg [18]. We selected as significant only probe sets with an adjusted *p*-value < 0.01 and an abs (log_2_ratio) equal to or larger than1 (which corresponds to a two-fold change in expression) to investigate further.

### Tissue specimens

Pancreatic endocrine tumour specimens were obtained from biobanks at the Department of Endocrine Oncology, the Department of Surgery, and the Department of Pathology at the Uppsala University Hospital. Frozen or paraffin embedded tissues were used. Tumours were initially frozen in liquid nitrogen and stored at -80°C until analysis. Inclusions were based on the availability of operative tissue specimens or biopsy material. Altogether two gastrinomas, two glucagonomas (one liver metastasis), five insulinomas and 14 non-functioning tumours were investigated. The mean age at diagnosis was 48 years (range 19-86). Seven tumours were from MEN 1 patients. The tumours were classified according to the WHO classification of endocrine neoplasms. For comparison, eight specimens of macroscopically determined non-tumourous pancreas adjacent to a pancreatic endocrine tumour were assessed by immunohistochemistry.

### Immunohistochemistry

Twenty-two PETs were immunostained for DKK1. Frozen, acetone-fixed sections (6 μm) were incubated with an anti-DKK1 rabbit polyclonal antibody (SC-25516, Santa Cruz Biotechnology) diluted in PBS with 1% BSA. The reaction product was revealed using a biotinylated secondary antibody, Vectastain Elite ABC, (Vector) and the chromogen 3-amino-9-ethylcarbazol and 0.02% hydrogen peroxide as a substrate. Sections were counterstained with Mayer's haematoxylin and mounted. Twelve paraffin embedded PET specimens were immunostained for TPH1. The rehydrated sections were heat-retrieved and incubated with an anti-TPH1 mouse antibody (Sigma Aldrich). The reaction product was revealed using the EnVision system -HPR (DakoCytomation, Copenhagen, Denmark), and DAB as the chromogen. Sections were counterstained with Mayer's haematoxylin and mounted. Each PET specimen and non-tumourous pancreatic specimens were evaluated independently by the authors and graded as low, high or heterogeneous (*i.e*. areas of both low and high expression present in the tumour). Immunostaining for ASCL1 has been published previously [8] and was graded as negative (-), weak (+), moderate (++), or strong (+++). In the present study we denoted strong (+++) staining in the cytoplasm as High and weak or moderate (+/++) as Low. Sections were photographed by an AxioCam MR camera employing the Axiovision imaging software using a LD A-plan 20×/40× 0.30 Ph1 objectives and a Zeiss Axiovert 40 microscope (Carl Zeiss Inc.).

### Statistical analysis

Unpaired *t *test was used for calculations regarding qPCR expression. A *p*-value below 0.05 was considered significant.

### Ethical approval

Permission for this study was obtained from the Uppsala Ethical Committee, Sweden. Informed consent was gathered from all patients.

## Results

### Expression profiling in the pancreatic endocrine tumour cell line BON1

**Figure 1:**
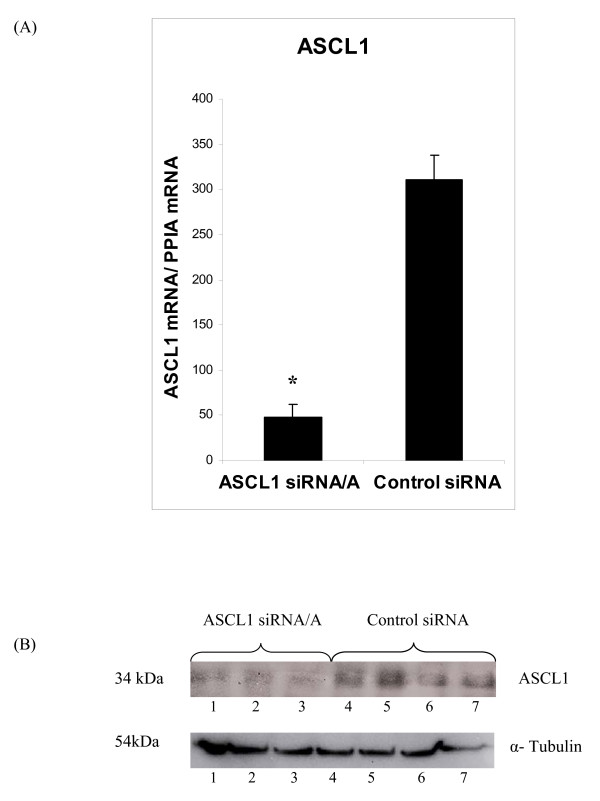
**(A) Relative expression of *ASCL1 *in siRNA-transfected BON1 cells as determined by qPCR. Expression levels were normalised to *ACTB *and *PPIA *with similar results**. Data are presented as mean ± SEM of analysis in triplicates. * *p *< 0.05. (B) Western blotting analysis for ASCL1. Protein extracts from siRNA-transfected BON1 cells as indicated.

**Figure 2:**
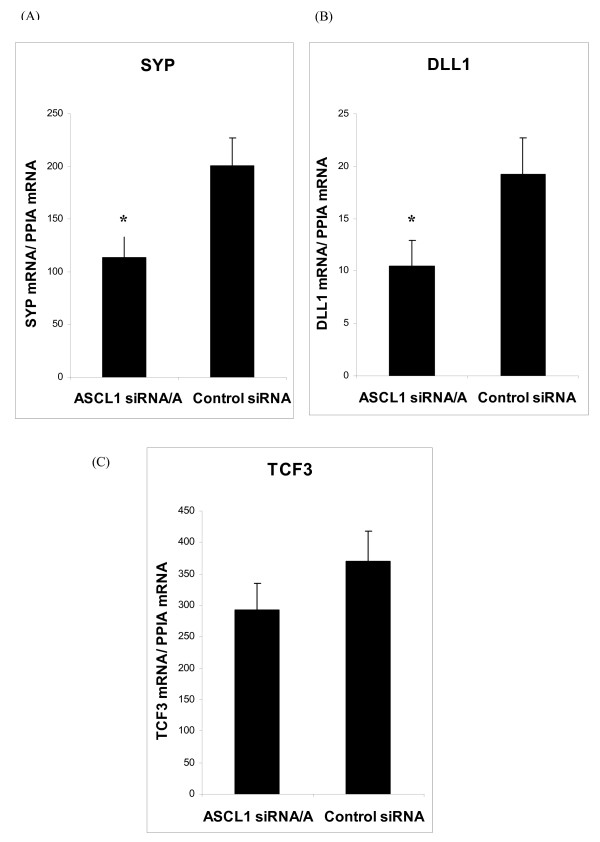
**Relative expression of *SYP*, *DLL1*, and *TCF3 *in siRNA-transfected BON1 cells as determined by qPCR**. Expression levels were normalised to *ACTB *and *PPIA *with similar results. Data are presented as mean ± SEM of analysis in triplicates. * *p *< 0.05.

RNA interference and microarray expression analysis were employed in order to identify *ASCL1 *target genes in BON1 cells. A specific siRNA to *ASCL1 *(*ASCL1 *siRNA/A) and one non-specific Control siRNA were transfected to BON1 cells. These cells are notoriously difficult to transfect and 30 nmol of siRNA was found to be optimal. *ASCL1 *siRNA/A was found to significantly (*p *< 0.0001) reduce *ASCL1 *mRNA expression compared to Control siRNA (Figure 1A). Importantly, ASCL1 protein expression was similarly reduced (Figure 1B). In order to further validate the experimental system for microarray expression analysis, the effects of reduced ASCL1 expression by RNAi was evaluated on the known or putative ASCL1 target genes Delta 1 (*DLL1*) and Synaptophysin (*SYP*). ASCL1 is known to bind to the DLL1 promoter and synergistically activate transcription together with Pou3f3 and Pou3f4 [19,20]. Reduced expression of ASCL1 negatively affects *SYP *expression in SCLC cells as well as in pulmonary endocrine cells of Ascl1 double null mice [3]. As a putative negative control we also assessed expression of the transcription factor *TCF3 *(*E12/E47*); a recognised dimerisation partner of ASCL1 that is required for transcription activation of ASCL1 target genes [21,22]. The results showed that siRNA/A to *ASCL1 *significantly reduced *DLL1 *(*p *= 0.001) and *SYP *(*p *= 0.01) expression, while expression of *TCF3 *was unaffected (Figures 2A-C).

**Figure 3:**
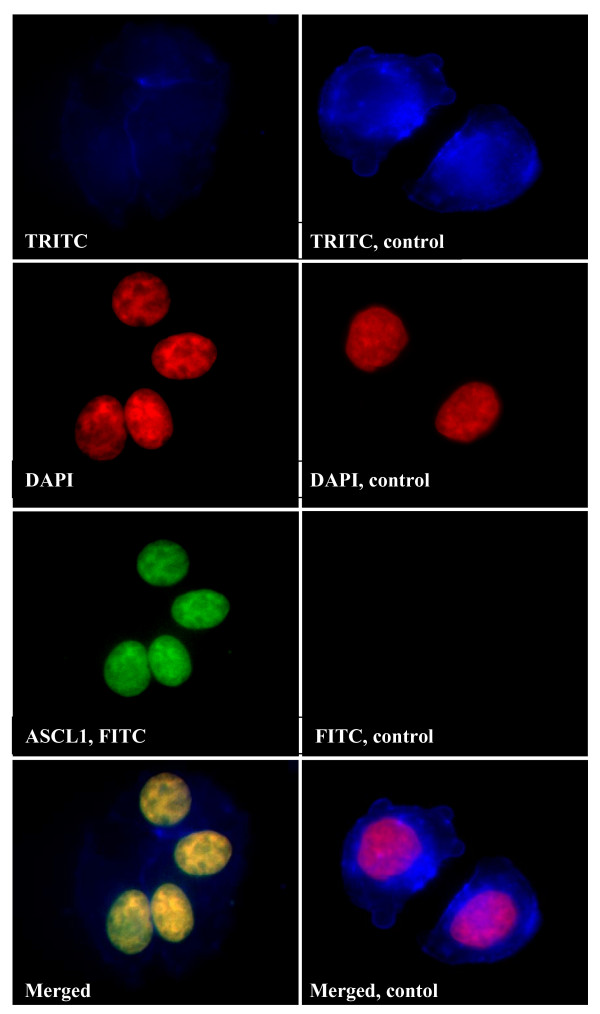
**Fluorescent immunostaining of ASCL1 in BON1 cells**. Cells were visualised by TRITC-labeled phalloidin (blue) and DAPI (red). Primary antibodies to ASCL1 were detected by FITC-labelled secondary antibodies (green). Yellow, indicates co-localisation (merged).

**Figure 4:**
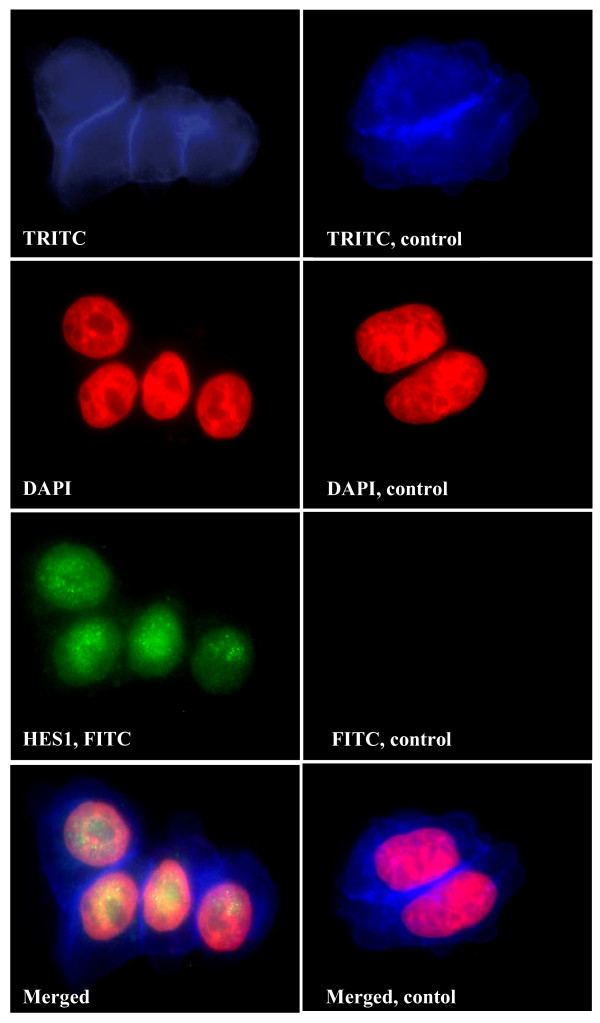
**Fluorescent immunostaining of HES1 in BON1 cells. Cells were visualised by TRITC-labeled phalloidin (blue) and DAPI (red)**. Primary antibodies to HES1 were detected by FITC-labelled secondary antibodies (green). Yellow, indicates co-localisation (merged).

Since we have observed lack of nuclear HES1 in PETs [8], protein expression in BON1 cells was investigated by fluorescent immunostaining. ASCL1 and HES1 were clearly expressed in BON1 cells, with prominent nuclear association (Figures 3 and 4).

**Figure 5:**
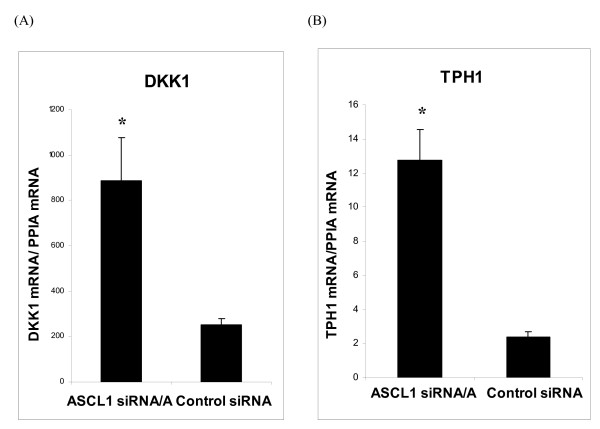
**Relative expression of *DKK1*and *TPH1 *in siRNA-transfected BON1 cells as determined by qPCR**. Data are presented as mean ± SEM of analysis in triplicates. * *p *< 0.05.

Six validated RNA samples from transfections with *ASCL1 *siRNA/A and Control siRNA were selected for microarray expression analysis employing the Human Genome U133 Plus 2.0 GeneChip. A total of 433 transcripts showed at least a two-fold difference in expression in BON1 cells transfected with *ASCL1 *siRNA compared to Control siRNA. Among annotated genes, 46 showed increased- and 112 reduced expression (Table 1; Table 2). As anticipated, *ASCL1 *expression was decreased (Table 2). Expression of both *DKK1 *and *TPH1 *were found to be increased in *ASCL1 *siRNA transfected cells (Table 1; Figure 5). Thus, *ASCL1 *negatively regulates *DKK1 *and *TPH1 *in BON1 pancreatic endocrine tumour cells. Other *ASCL1 *target genes included oncogenes (like *MYCN *and *RET*), those involved in the integrin system (*NRXN3*, *LAMA4 *and *SMOCK2*), participating in apoptosis (*PDCD6, CFLAR *and *CCAR1*), as well as genes known to be involved in the Notch, Wnt, NFκβ, TGFβ and MAP kinas signalling pathways. Many of the *ASCL1 *targets represent potential oncogenes and tumour suppressor genes.

**Table 1:** Annotated genes with increased expression in BON1 cells transfected with siRNA to *ASCL1*

Gene Symbol	Gene Name	Location	Ratio	adj p Value
*RBM24*	RNA binding motif protein 24	6p22.3	4,708	0,0000391
*HAS2*	hyaluronan synthase 2	8q24.12	3,279	0,0002805
*MYCN*	v-myc myelocytomatosis viral related oncogene,	2p24.1	2,777	0,0000305
*C13orf15*	chromosome 13 open reading frame 15	13q14.11	2,594	0,0000701
*APCDD1*	adenomatosis polyposis coli down-regulated 1	18p11.22	2,569	0,0000305
*LGR5*	leucine-rich repeat-containing G protein-coupled receptor 5	12q22-q23	2,540	0,0003188
DKK1*	dickkopf homolog 1 (Xenopus laevis)	10q11.2	2,349	0,0000155
TPH1*	Tryptophan hydroxylase 1	11p15.3-p14	2,325	0,0002805
*ID4*	Inhibitor of DNA binding 4	6p22-p21	2,317	0,0001962
*PVRL3*	poliovirus receptor-related 3	3q13	2,295	0,0000949
*TIMP2*	TIMP metallopeptidase inhibitor 2	17q25	2,286	0,0000200
*ASAM*	adipocyte-specific adhesion molecule	11q24.1	2,254	0,0002763
*SPOCK1*	sparc/osteonectin, cwcv and kazal-like domains	5q31	2,251	0,0000796
*NRXN3*	neurexin 3	14q31	2,246	0,0002025
*KL*	klotho	13q12	2,240	0,0000150
*GPD2*	glycerol-3-phosphate dehydrogenase 2 (mitochondrial)	2q24.1	2,238	0,0000155
*CLIC5*	chloride intracellular channel 5	6p21.1-p12.1	2,235	0,0000313
*EMG1*	EMG1 nucleolar protein homolog (S. cerevisiae)	12p13	2,230	0,0000489
*DRD1IP*	dopamine receptor D1 interacting protein	10q26.3	2,219	0,0001121
*CXCR7*	chemokine (C-X-C motif) receptor 7	2q37.3	2,216	0,0000862
*STOML3*	stomatin (EPB72)-like 3	13q13.3	2,196	0,0003867
*SI*	sucrase-isomaltase (alpha-glucosidase)	3q25.2-q26.2	2,181	0,0000701
*FGF13*	fibroblast growth factor 13	Xq26.3	2,178	0,0000288
*CHM*	choroideremia (Rab escort protein 1)	Xq21.2	2,150	0,0000396
*GLCE*	glucuronic acid epimerase	15q23	2,144	0,0000561
*DC2*	DC2 protein	4q25	2,143	0,0000181
*SMS*	spermine synthase	Xp22.1	2,113	0,0003207
*GPD2*	glycerol-3-phosphate dehydrogenase 2 (mitochondrial)	2q24.1	2,095	0,0000586
*LOC283454*	hypothetical protein LOC283454	12q24.23	2,095	0,0000465
*CXorf57*	chromosome X open reading frame 57	Xq22.3	2,094	0,0003208
*PTGES3*	prostaglandin E synthase 3 (cytosolic)	12.	2,091	0,0000430
*HS2ST1*	heparan sulfate 2-O-sulfotransferase 1	1p31.1-p22.1	2,080	0,0001750
*EMP1*	epithelial membrane protein 1	12p12.3	2,066	0,0000357
*EBAG9*	estrogen receptor binding site associated, antigen, 9	8q23	2,058	0,0000919
*VPS37B*	vacuolar protein sorting 37 homolog B (S. cerevisiae)	12q24.31	2,049	0,0000640
*NOV*	nephroblastoma overexpressed gene	8q24.1	2,001	0,0000746

**Table 2:** Annotated genes with decreased expression in BON1 cells transfected with siRNA to *ASCL1*

Gene Symbol	Gene Name	Location	Ratio	adj P Value
*FAM87A*	family with sequence similarity 87, member A	8p23.3	0,221	0,0000104
*DKFZP761C1711*	Hypothetical protein DKFZp761C1711		0,246	0,0000258
*GABRA1*	gamma-aminobutyric acid (GABA) A receptor, alpha 1	5q34-q35	0,260	0,0000172
*GUSBP1*	glucuronidase, beta pseudogene 1	7q21.11	0,277	0,0000104
*TncRNA*	trophoblast-derived noncoding RNA	11q13.1	0,284	0,0000104
*LOC728411*	Similar to Beta-glucuronidase precursor	5.	0,296	0,0000104
*TTLL5*	tubulin tyrosine ligase-like family, member 5	17q21.32	0,328	0,0001110
*EBF1*	Early B-cell factor 1	5q34	0,333	0,0000112
*VPS13C*	Vacuolar protein sorting 13 homolog C (S. cerevisiae)	15q21.3	0,341	0,0000150
*RNF12*	Ring finger protein 12	Xq13-q21	0,342	0,0000283
ASCL1*	achaete-scute complex homolog 1 (Drosophila)	12q22-q23	0,351	0,0000162
*SORBS2*	sorbin and SH3 domain containing 2	4q35.1	0,353	0,0000161
*ERBB4*	v-erb-a erythroblastic leukemia viral oncogene homolog 4	2q33.3-q34	0,359	0,0001092
*FLJ38379*	hypothetical protein FLJ38379	2q37.3	0,360	0,0001245
*TCF12*	Transcription factor 12	15q21	0,365	0,0000283
*PGM5*	phosphoglucomutase 5	9q13	0,365	0,0000499
*RIT1*	Ras-like without CAAX 1	1q22	0,366	0,0000150
*ZCCHC7*	Zinc finger, CCHC domain containing 7	9p13.2	0,371	0,0001730
*LOC730168*	hypothetical protein LOC730168///LOC732289	3q26.32	0,374	0,0000625
*CFLAR*	CASP8 and FADD-like apoptosis regulator	2q33-q34	0,374	0,0001083
*C11orf80*	chromosome 11 open reading frame 80	11q	0,375	0,0000579
*CCAR1*	Cell division cycle and apoptosis regulator 1	10q21.3	0,375	0,0000796
*FAM81B*	family with sequence similarity 81, member B	5q15	0,382	0,0000599
*FLJ25770*	hypothetical protein FLJ25770	4q21.1	0,383	0,0000181
*LOC730390*	SMA4///similar to SMA4	5q13	0,385	0,0000159
*ZFAND6*	Zinc finger, AN1-type domain 6	15q25.1	0,387	0,0000246
*LOC728555*	hypothetical protein LOC728555///LOC730391	5q13.2	0,388	0,0000151
*GRAMD3*	GRAM domain containing 3	5q23.2	0,394	0,0000136
*CBFA2T2*	core-binding factor, runt domain, alpha subunit 2	20q11	0,394	0,0000181
*ZNF638*	Zinc finger protein 638	2p13.2-p13.1	0,396	0,0019556
*LOC728678*	hypothetical protein LOC728678///LOC731914	3p22.3	0,397	0,0000150
*CLCN5*	chloride channel 5	Xp11.23-p11.22	0,398	0,0000170
*ANXA13*	annexin A13	8q24.13	0,412	0,0000181
*RP11-506K6.3*	Hypothetical LOC389362	6p25.2	0,415	0,0002632
*FLJ23556*	hypothetical protein FLJ23556	10q25.2	0,417	0,0000788
*PFAAP5*	Phosphonoformate immuno-associated protein 5	13q13.1	0,417	0,0000150
*PCDHGA4*	protocadherin gamma subfamily A, 4	5q31	0,419	0,0000274
*LOC145474*	hypothetical protein LOC145474	14q24.1	0,421	0,0003892
*IFIT1*	interferon-induced protein	10q25-q26	0,421	0,0006010
*DKFZp547E087*	hypothetical gene LOC283846	18p11.21	0,424	0,0000586
*TXNIP*	thioredoxin interacting protein	1q21.1	0,427	0,0000305
*CDC14B*	CDC14 cell division cycle 14 homolog B	9q22.33	0,432	0,0000885
*RUFY2*	RUN and FYVE domain containing 2	10q21.3	0,432	0,0001373
*KLHL28*	Kelch-like 28 (Drosophila)	14q21.3	0,435	0,0002095
*MBNL2*	Muscleblind-like 2 (Drosophila)	13q32.1	0,436	0,0000246
*LOC730496*	hypothetical protein LOC730496	1.	0,436	0,0000150
*PRKAA1*	protein kinase, AMP-activated, alpha 1 catalytic subunit	5p12	0,436	0,0002228
*NR5A2*	nuclear receptor subfamily 5, group A, member 2	1q32.1	0,438	0,0003777
*LOC388743*	similar to calpain 8	1q41	0,440	0,0000284
*PTPN13*	Protein tyrosine phosphatase, non-receptor type 13	4q21.3	0,441	0,0011559
*RASAL2*	RAS protein activator like 2	1q24	0,442	0,0003715
*LOC730258*	neuroblastoma breakpoint family, member 1, 3, 8, 10	1q21.1	0,443	0,0000150
*ZNF518*	Zinc finger protein 518	10q23.33	0,445	0,0002293
*SCNN1A*	sodium channel, nonvoltage-gated 1 alpha	12p13	0,445	0,0000176
*CTAGE5*	CTAGE family, member 5	14q13.3	0,446	0,0000617
*LOC440895*	similar to LIM and senescent cell antigen-like domains 3	2q13	0,446	0,0003164
*PELI1*	Pellino homolog 1 (Drosophila)	2p13.3	0,449	0,0001121
*FAM98A*	Family with sequence similarity 98, member A	2p22.3	0,449	0,0000499
*BRWD2*	bromodomain and WD repeat domain containing 2	10q26	0,450	0,0000602
*C20orf74*	chromosome 20 open reading frame 74	20p11.22	0,450	0,0000926
*MALAT1*	metastasis associated lung adenocarcinoma transcript 1	2p16.3	0,452	0,0001060
*RTN4*	reticulon 4	2p16.3	0,453	0,0004446
*LOC654342*	Similar to lymphocyte-specific protein 1	2p11.1	0,454	0,0000284
*CYorf15B*	chromosome Y open reading frame 15B	Yq11.222	0,455	0,0001245
*SMOC2*	SPARC related modular calcium binding 2	6q27	0,456	0,0002821
*CAPN2*	calpain 2, (m/II) large subunit	1q41-q42	0,457	0,0000460
*HEL308*	DNA helicase HEL308	4q21.23	0,459	0,0006091
*PDCD6*	Programmed cell death 6	5pter-p15.2	0,459	0,0000722
*LOC285147*	hypothetical protein LOC285147	2p25.2	0,460	0,0001063
*TFF3*	trefoil factor 3 (intestinal)	21q22.3	0,460	0,0010306
*RIMBP2*	RIMS binding protein 2	12q24.33	0,461	0,0000520
*C10orf93*	chromosome 10 open reading frame 93	10q26.3	0,462	0,0000950
*SUCNR1*	succinate receptor 1	3q24-q25.1	0,462	0,0000603
*LOC151878*	hypothetical protein LOC151878	3p14.3	0,463	0,0003715
*LRRFIP1*	Leucine rich repeat (in FLII) interacting protein 1	2q37.3	0,465	0,0000339
*ADAM12*	ADAM metallopeptidase domain 12 (meltrin alpha)	10q26.3	0,465	0,0003076
*GART*	Phosphoribosylglycinamide, phosphoribosylaminoimidazole	21q22.11	0,466	0,0003142
*PLGLB1/2*	plasminogen-like B2///plasminogen-like B1	2p11-q11	0,467	0,0007445
*RET*	ret proto-oncogene	10q11.2	0,467	0,0000247
*POLQ*	polymerase (DNA directed), theta	3q13.33	0,467	0,0002632
*KIAA1632*	KIAA1632	18q12.3-q21.1	0,468	0,0004005
*ADAM28*	ADAM metallopeptidase domain 28	8p21.2	0,469	0,0000950
*MSI2*	Musashi homolog 2 (Drosophila)	17q22	0,469	0,0000344
*JMJD1C*	jumonji domain containing 1C	10q21.2-q21.3	0,470	0,0001013
*DST*	dystonin	6p12.1	0,473	0,0001509
*NT5E*	5'-nucleotidase, ecto (CD73)	6q14-q21	0,474	0,0000288
*LYST*	lysosomal trafficking regulator	1q42.1-q42.2	0,475	0,0001027
*SYK*	Spleen tyrosine kinase	9q22	0,476	0,0003933
*DLG1*	Discs, large homolog 1 (Drosophila)	3q29	0,476	0,0006683
*RASSF6*	Ras association (RalGDS/AF-6) domain family 6	4q13.3	0,476	0,0006133
*TRA2A*	transformer-2 alpha	1p36.11	0,478	0,0000701
*UBE2D3*	ubiquitin-conjugating enzyme E2D 3	4q24	0,479	0,0002430
*TMEM46*	transmembrane protein 46	13q12.13	0,480	0,0000248
*INADL*	InaD-like (Drosophila)	1p31.3	0,480	0,0002693
*TTC30A*	tetratricopeptide repeat domain 30A	2q31.2	0,484	0,0003577
*SNAP25*	Synaptosomal-associated protein, 25 kDa	20p12-p11.2	0,484	0,0000189
*PRO2852*	hypothetical protein PRO2852	9.	0,485	0,0000460
*MLLT3*	myeloid/lymphoid or mixed-lineage leukemia	9p22	0,486	0,0004334
*RBM6*	RNA binding motif protein 6	3p21.3	0,488	0,0002432
*PPP2R5C*	protein phosphatase 2, regulatory subunit B',	14q32	0,488	0,0000241
*GOPC*	Golgi associated PDZ and coiled-coil motif containing	6q21	0,489	0,0006453
*LAMA4*	laminin, alpha 4	6q21	0,489	0,0001402
*SFRS15*	splicing factor, arginine/serine-rich 15	21q22.1	0,490	0,0000344
*KIF13A*	kinesin family member 13A	6p23	0,491	0,0001644
*CLASP2*	cytoplasmic linker associated protein 2	3p22.3	0,493	0,0004942
*MMAA*	Methylmalonic aciduria (cobalamin deficiency) cblA type	4q31.22	0,493	0,0002644
*C1orf192*	chromosome 1 open reading frame 192	1q23.3	0,494	0,0006859
*hCG_2003663*	hCG2003663	9q22.32	0,494	0,0006724
*SMOC1*	SPARC related modular calcium binding 1	14q24.2	0,494	0,0001073
*REV3L*	REV3-like, catalytic subunit of DNA polymerase zeta	6q21	0,496	0,0001092
*SMAD1*	SMAD family member 1	4q31	0,496	0,0004345
*TWF1*	twinfilin, actin-binding protein, homolog 1 (Drosophila)	12q12	0,497	0,0001687
*FBXO9*	F-box protein 9	6p12.3-p11.2	0,497	0,0000391

Gene ontology (GO) were applied to identify the functional significance of all (n = 433) differentially expressed transcripts with known function(s) http://www.geneontology.org. Each differentially expressed transcript was placed in functional GO categories and over-represented categories are shown. The enrichment of the GO data was narrowed down to broad GO terms. The division was based on biological process, molecular function, and cellular components. The most over-represented GO biological process categories, according to number of involved transcripts, related to regulation of a biological or cellular processes, development, metabolic processes or transcription and regulation of transcription. For molecular function, most over-represented categories were binding activity (receptor, DNA or nucleic acid) and transcription regulation (cofactor or binding activity). For cellular components, the most over-represented category was transcripts involved in cellular junctions (Table 3).

**Table 3:** Functional GO categories

Systems	Categorys	No. of genes or transcripts
Biological processes		
	Regulation of biological or cellular process	342
	Development regulation/cellular	292
	Cellular metabolic process	189
	Transcription and regulation of transcription	144
	Biological/cellular adhesion	68
	Regulation of nucleo -base -side, -tide and nucleic acid metabolic process	62
	Regulation of gene expression	56
	Cell differentiation	44
	Locomotion, cellular or regulation of	29
	Cellular migration/localisation	26
	Phosphorylation	20
	Neurogenes	19
	Protein amino acid phosphorylation	17
	Carbohydrate biosynthetic process	5
	Integrin-mediated signalling pathway	5
Molecular functions		
	Binding activity, receptor, DNA, nucleic acid	305
	Transcription regulation/cofactor or binding activity	67
	Kinase activity	54
	Phosphotransferase activity	49
	Ligase activity ubiquitin/amino acid/small conjugating protein	24
	Ligase activity	20
	Enzyme activator activity	13
	Transmembrane receptor protein kinase activity	11
Cellular components		
	Cell junction	14
	Intercellular junction	8
	Basement membrane	5

Functional categories are based on GO annotation. Note that in GO function hierarchy, some genes/transcripts belong to multiple categories.

### Inverse expression of ASCL1 and DKK1 in the majority of investigated PETs

**Figure 6:**
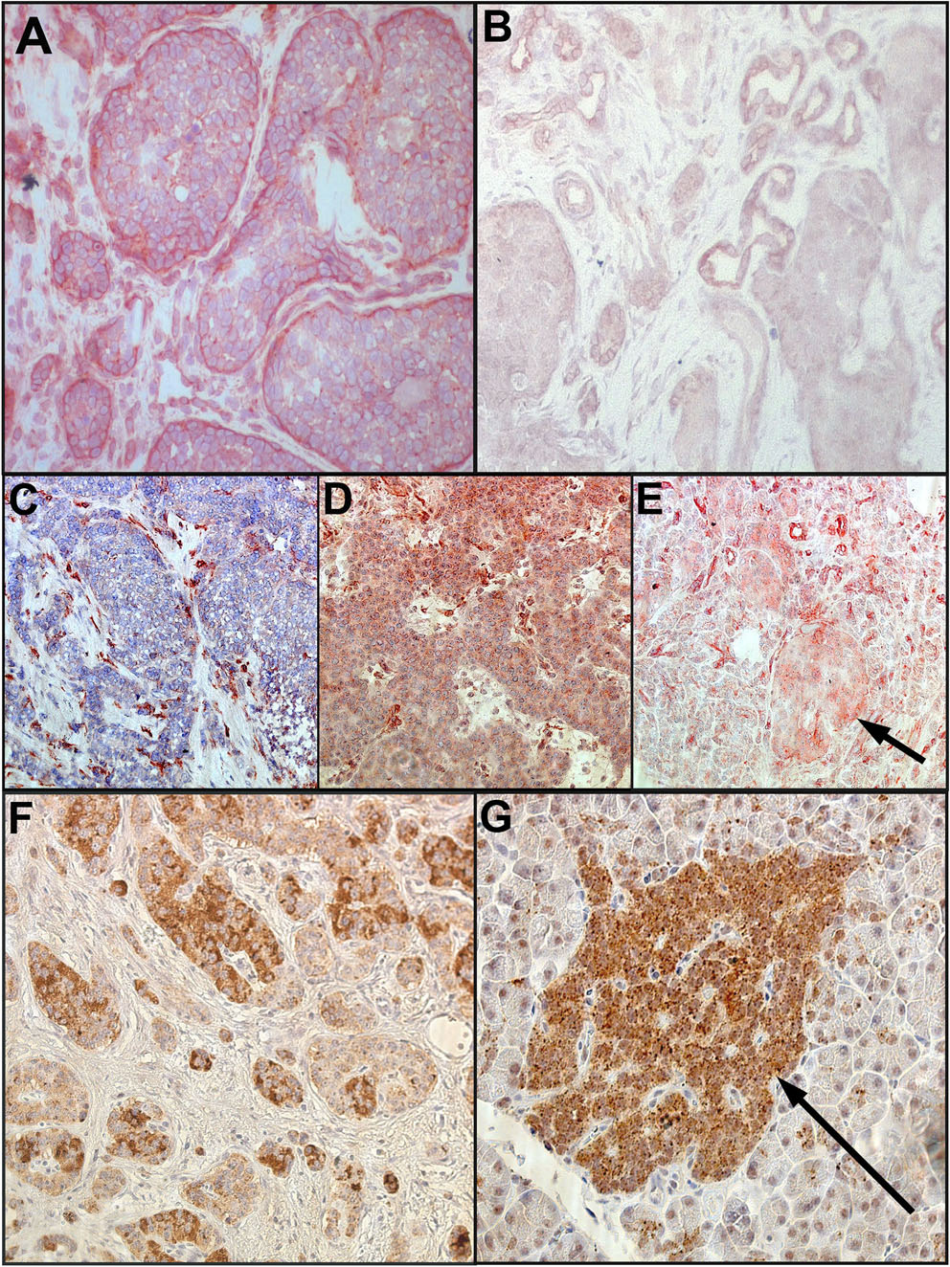
**Immunohistochemical analysis of ASCL1, DKK1, and TPH1 in pancreatic endocrine tumours and non-tumourous pancreatic specimen**. Pancreatic islets are indicated by arrows. Representative immunostainings are shown. (A) High ASCL1 expression in tumour no. 5 (×200), (B) Low ASCL1 expression in tumour no. 23 (×200), (C) Low DKK1 expression in tumour no. 5 (×200), (D) High DKK1 expression in tumour no. 10 (×200), (E) DKK1 expression in non-tumourous pancreas (×200), (F) heterogeneous TPH1 expression in tumour no. 10 (×200), (G) TPH1 expression in non-tumourous pancreas (×400).

Expression of DKK1 was evaluated by immunohistochemistry in 22 out of the 23 analysed PETs (Figure 6; Table 4). Inverse relation of ASCL1 [8] to DKK1 expression was observed for 15 out of 22 tumours (68%). Of these, nine tumours displayed low ASCL1/high DKK1 and six tumours high ASCL1/low DKK1 expression. Thus, ASCL1 is likely to negatively regulate DKK1 transcription in these tumours, as has been shown to occur in A549 lung cancer cells [9]. The remaining PETs showed high ASCL1/high DKK1 (n = 4) or low ASCL1/low DKK1 (n = 3) expression. No relations of ASCL1/DKK1 expression to tumour syndrome, MEN 1, or WHO classification were observed.

**Table 4:** Clinical characteristics and results of immunohistochemistry for ASCL1, DKK1 and TPH1

Tumour no	Gender	Age at diagnosis	WHO	Syndrome	ASCL1	DKK1	TPH1
1	M	53	2	NF	High	Low	Heterogeneous
2	F	47	1	NF/MEN 1	High	Low	Low
3	M	50	2	IN	High	Low	
4	F	86	1	IN	High	Low	
5	F	40	1	GA	High (p)	Low (p)	Heterogeneous
6	M	44	2	GL	High	Low	Heterogeneous
7	M	51	2	NF	Low	High	
8	F	46	2	NF	Low	High	
9	F	34	2	NF	Low	High	
10	M	62	1	NF/MEN 1	Low	High (p)	Heterogeneous (p)
11	M	53	2	NF/MEN 1	Low	High	
12	M	48	2	NF/MEN 1	Low	N/D	Heterogeneous
13	M	47	2	IN	Low	High	Heterogeneous
14	M	19	1	IN/MEN 1	Low	High	Heterogeneous
15*	M	53	2	GL	Low	High	High
16	M	72	2	GA	Low	High	
17	M	50	3	NF	High	High	
18	F	44	2	NF	High	High	
19	F	23	3	NF	High	High	
20	M	57	1	NF/MEN 1	High	High	High
21	F	44	1	NF	Low	Low	Heterogeneous
22	M	22	1	NF/MEN 1	Low	Low	High/Heterogeneous
23	M	64	1	IN	Low (p)	Low	

Immunoreactivity was graded as Low, High or Heterogeneous. ASCL1 immunoreactivity has been determined previously (8) were grading +, ++ is here denoted Low and +++ denoted High. N/D, not determined; *, liver metastasis; p, in Figure 6. WHO classifications: 1, well-differentiated endocrine tumour, 2, well-differentiated endocrine carcinoma, 3, poorly differentiated endocrine carcinoma. NF, non-functioning; MEN 1, Multiple Endocrine Neoplasia 1; IN, insulinoma; GA, gastrinoma; GL, glucagonoma

### TPH1 displays heterogeneous expression with no relation to ASCL1 in PETs

The amount of immunoreactivity varied for TPH1. Nine out of the twelve analysed PETs (75%) showed a heterogeneous expression pattern (Figure 6F, Table 4). High expression was seen in two tumours and low expression in one. Tumours with high or heterogeneous expression showed a somewhat lower TPH1 expression than control non-tumourous pancreatic tissue. No relations of ASCL1 to TPH1 expression or to clinical characteristics were observed.

## Discussion

This study showed altogether 433 target transcripts (158 annotated genes) in the human pancreatic endocrine tumour cell line BON1 that directly or indirectly were regulated by *ASCL1*, among them several putative oncogenes and suppressor genes. *ASCL1 *was found to negatively regulate *DKK1 *and *TPH1 *expression in BON1 cells. This may suggest that Notch1 signalling pathway regulatory factor(s) other than ASCL1 is involved in the reduced expression of *TPH1 *observed in Notch1 overexpressing BON cells [12]. In order to investigate if this relation between *ASCL1*, *DKK1 *and *TPH1 in vitro *might be of relevance *in vivo*, we analysed their protein expression in PETs. An inverse relation of ASCL1 to DKK1 expression was observed in 68% of the analysed tumours (n = 22). No obvious relation between ASCL1 and TPH1 expression levels was found.

ASCL1 has been found to repress DKK1 transcription, a negative regulator of the Wnt signalling pathway in lung cancer cells, and is also the first transcriptional repressor identified for DKK1. The regulation is meditated by histone deacetylation and repressive lysine 27 trimetylation in the promoter region of DKK1 [9]. Moreover, downregulation of DKK1 has been associated with colorectal- and breast cancer (23, 24). On the other hand, DKK1 has also been identified as a potential prognostic and diagnostic marker for cohorts of breast cancer patients with poor prognosis [23] and increased circulating levels of DKK1 has been associated with the presence of bone metastases in patients with breast cancer [25] We note that 13 out of the 22 analysed PETs prominently expressed DKK1.

Wnt/β-catenin signalling is negatively regulated by DKK1 by inhibition of the complex formation between Wnts and its receptors, LRP5/6. It has been advocated that ASCL1 expression may favour cancer cell growth through repression of DKK1 with the consequential aberrant activation of the Wnt/β-catenin signalling pathway [9]. This may also apply to a subset of PETs as a total of 9 out of 22 PETs displayed low DKK1 immunoreactivity.

ASCL1 may have a coordinating role in production of serotonin by transcriptional regulation of TPH1 and could thereby be involved in causing the carcinoid syndrome in patients with PET [12]. Our results from the microarray expression analysis in BON1 cells suggested that TPH1 might constitute a ASCL1 target gene in BON1 cells. However, an obvious relation between ASCL1 and TPH1 protein expression levels were not found, and TPH1 showed a heterogeneous pattern of immunoreactivity in PETs.

Traditionally, much of the Notch signalling research has focused on the involvement of Notch signalling factors like ASCL1 in neural stem cell differentiation. Even though pancreatic endocrine cells have an endodermal origin they also share several molecular features with neurons. Like neurons in the central nervous system, differentiating endocrine cells in the pancreas appear in a scattered fashion within a field of progenitor cells. The different cell types are generated by lateral inhibition through Notch signalling [4]. With this in mind it is not surprising that the results from the GO analysis suggest that *ASCL1 *target genes participate in cellular differentiation, migration and localisation of cells also in pancreatic endocrine cells.

## Conclusion

The present findings support the notion that ASCL1 is involved in pancreatic endocrine tumourigenesis, where aberrant expression of DKK1 may play additional important roles. ASCL1 also directly or indirectly regulates expression of several putative oncogenes and tumours suppressor genes in pancreatic endocrine tumour cells that may contribute to the neoplastic process.

## Competing interests

The authors declare that they have no competing interests.

## Authors' contributions

TAJ performed the experiments. TAJ, GW and BS participated in design of the study, interpreted the result and contributed to writing the paper. All authors read and approved the final version of the manuscript.

## Pre-publication history

The pre-publication history for this paper can be accessed here:

http://www.biomedcentral.com/1471-2407/9/321/prepub
